# Higher bodily adiposity, fat intake, and cholesterol serum levels are associated with higher disease activity in psoriatic arthritis patients: is there a link among fat and skin and joint involvement?

**DOI:** 10.1186/s12944-020-1200-7

**Published:** 2020-02-07

**Authors:** Beatriz Figueiredo Leite, Melissa Aparecida Morimoto, Carina Gomes, Barbara Nascimento de Carvalho Klemz, Patrícia de Souza Genaro, Nágila Raquel Teixeira Damasceno, Vera Lúcia Szejnfeld, Marcelo de Medeiros Pinheiro

**Affiliations:** 1 Federal University of Sao Paulo (UNIFESP/ EPM). Rheumatology Division, 204 Leandro Dupré St., Room 74, Vila Clementino, Sao Paulo, 04025-010 Brazil; 2 Vale do Paraiba University, 2911 Shidhima Hifumi, Avenue.Urbanova, Sao Jose dos Campos, 12244-000 Brazil; 3 Sao Paulo University. School of Public Health. Nutrition Department, 715, Dr Arnaldo Avenue, Cerqueira César, Sao Paulo, 01246-904 Brazil

**Keywords:** Psoriatic arthritis, Body composition measurements, Adipose tissue, Metabolic syndrome, Food intake

## Abstract

**Introduction/ objectives:**

Assuming that there is a link between lipid and glucose metabolism and inflammation in patients with psoriatic arthritis (PsA), our aim was to evaluate the relationships among body composition measurements, food intake, and disease activity in patients with PsA.

**Methods:**

A total of 97 patients with PsA, according to the CASPAR criteria, were included in this cross-sectional study. Body composition measurements (whole-body DXA, GE-Lunar), food intake (3-day registry) and biochemical and inflammatory serum markers were evaluated. Skin and joint disease activity were assessed by using PASI, BSA, DAS28, and minimal disease activity (MDA). The level of significance was set as *p* < 0.05.

**Results:**

A higher prevalence of obesity, according to the fat mass index (FMI) (92.7%), and metabolic syndrome (MetS) (54%) were found, but no significant changes regarding lean or bone mass were found. Joint disease activity was positively correlated with total body fat (r = 0.4; *p* < 0.001), FMI (r = 0.33; *p* < 0.001), body mass index (r = 0.20; *p* < 0.049) and waist circumference (r = 0.27; *p* = 0.009). In addition, joint disease activity was negatively associated with muscle mass (r = − 0.38; p < 0.001). Skin disease activity was positively correlated with total cholesterol (r = 0.3; *p* = 0.003) and LDL-cholesterol (r = 0.28; *p* = 0.006). After multiple adjustments, patients with severe joint disease activity had higher body adiposity than patients in remission or with low disease activity. Skin disease activity was associated with higher trans-fat intake and lower omega-6 consumption.

**Conclusions:**

Our data suggest a possible harmful link among fat (body adiposity, saturated fat consumption, LDL-cholesterol serum levels) and joint and skin disease activity in patients with PsA.

## Introduction

Psoriatic arthritis (PsA) is a chronic systemic inflammatory disease characterized by red scaly skin patches and nail and joint involvement is associated with multiple comorbidities, particularly metabolic syndrome (MetS) [1–3], characterized by higher obesity and body adiposity [4] and poor lipid profiles [2, 5]. This close relationship between adipose tissue and skin and joint disease may be explained by complex interactions among inflammation, innate immune changes, insulin uptake, lipid processing, and alterations in adipogenesis and neoangiogenesis [6–8].

Some authors have found significant association between body mass index (BMI) and disease activity in psoriasis (Ps) and PsA patients [9–13], especially in patients with a longer duration of disease [14]. Moreover, better response to cyclosporine was observed in obese patients with Ps with low calorie intake and restricted diet, suggesting that lifestyle modifications may contribute to the pharmacological therapy [9, 15]. Additioonally, higher carbohydrate and saturated fat intakes were associated with skin disease activity and a higher rate of comorbidities [16].

Assuming that there is a link between lipid and glucose metabolism and the inflammation in patients with PsA, our aim was to evaluate the relationship among body composition (BC) measurements, food intake, and disease activity in patients with psoriatic arthritis (PsA).

## Patients and methods

**Fig. 1:**
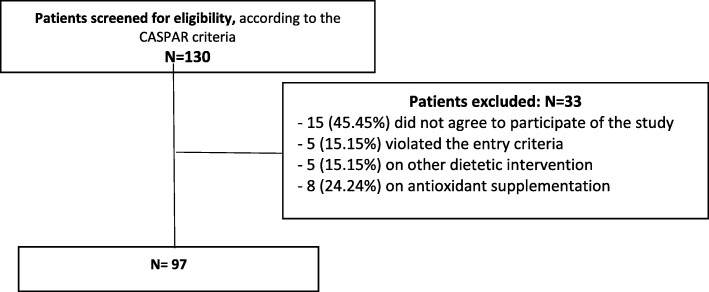
Patient disposition. Intent to treat analysis. CASPAR: ClAssification of psoriatic arthritis

A total of 97 patients were included in this cross-sectional study (Fig. 1).

As an inclusion criterion, patients with PsA must have been diagnosed according to the Classification Criteria of Psoriatic Arthritis (CASPAR) and must have signed an informed consent form, according to the Declaration of Helsinki. Specific medications for PsA and physical activity levels were required to be stable for the last 3 months. Patients with gastrointestinal, endocrine, pulmonary, kidney, hepatic, and neuromuscular diseases, as well as patient who were HIV-positive, pregnant or breast-feeding or had a previous history of cancer were excluded. Patients taking sex steroid hormones, protein supplements, vitamins, multivitamins, nutraceuticals or antioxidants were not included.

Clinical risk factors for MetS and CVD were evaluated in all PsA patients. To classify MetS, the Harmonizing Guideline for Metabolic Syndrome was used [17]. The criteria for clinical diagnosis were the presence of any three of five risk factors: 1) elevated waist circumference, according to population and country specific definitions; 2) elevated triglycerides (≥ 150 mg/dL or 1.7 mmol/L) or drug treatment for elevated triglycerides; 3) reduced high-density lipoprotein cholesterol (HDL-C) (< 40 mg/dL or 1.0 mmol/L in males; < 50 mg/dL or 1.3 mmol/L in females); 4) elevated blood pressure (systolic ≥130 and/or diastolic ≥85 mmHg) or antihypertensive drug treatment; 5) elevated fasting glucose (≥ 100 mg/dL) or drug treatment for elevated glucose. In addition, medical history including current drug use, lifestyle habits, duration of disease, and details about skin and joint involvement were also recorded.

A 3-day food-record (FR) was used to quantify the intake of energy (kcal), carbohydrates (g), protein (g), total fat (g), saturated fat (g), monounsaturated fat (g), polyunsaturated fat (g), cholesterol (mg), trans-fat (g), sugar (g), fiber (g), vitamin E (mg), vitamin A (mcg), vitamin C (mg), magnesium (mg), zinc (mg), copper (mg), selenium (mg), omega 3 (ω-3) (g), omega 6 (ω-6) (g), carotene (RE), beta-carotene (mg), and sodium (mg). A well-trained dietitian prospectively administered these FR. Energy was adjusted using the residual method described by Willet and Stamper (1998) [18]. Data were calculated using *he Food Processor SQL – Professional Nutrition Analysis Software and Databases – ESHA Research, USA, 2010.* Dietetic data were compared with reference values, according to the Dietary Reference Intake (DRI) [19].

Anthropometric assessment was performed by measuring weight (Filizola®) and height (stadiometer). Nutritional status was categorized based on the World Health Organization (WHO) criteria for BMI (kg/ m^2^). Waist circumference was measured halfway between the lowest rib and the top of hipbone and was classified using a cutoff of 90 cm for males and 80 cm for females, values proposed by Ethnic Central and South American populations by the International Diabetes Federation (IDF) and Metabolic Syndrome Harmonizing Guidelines [17].

Body composition assessment was performed by using dual X-ray absorptiometry (DXA) technology (GE-Lunar Radiation Corporation, DPX MD +, Madison, WI, USA), according to the standard protocol for acquisition and analysis suggested by International Society Clinical Densitometry (ISCD). The measurements included total lean mass (kg), skeletal lean mass (kg), total and regional adipose tissue (kg and %), total bone mineral density (g/ cm^2^), and bone mineral content (g). The coefficients of variation were 1.14, 1.64, 1.53, 1.62, 0.67, and 1.72%, respectively [20]. To classify low appendicular lean mass (ALM), Baumgartner’s method was used for patients older than 50 years, and Rosetta’s method was used for those under 50 years, according to sex [21, 22]. The fat mass index (FMI) was calculated using the equation proposed by NHANES III, considering reference values of 5–9 kg/ m^2^ for females and 3–6 kg/ m^2^ for males [23].

To evaluate the activity and severity of skin disease, the psoriasis area severity index (PASI) [24] and body surface area (BSA) [25] were used. To evaluate peripheral joint activity, the disease activity score (DAS28-ESR and DAS28-CRP) (interobserver coefficient of variation of 0.81 and intraobserver coefficient of variation of 0.79) [26] were used. For axial involvement, the Bath Ankylosing Spondylitis disease activity index (BASDAI) was chosen [27]. The functional capacity was evaluated using the health assessment questionnaire (HAQ) [28] and minimal disease activity (MDA) was used to classify remission status in PsA patients [29].

Physical activity status was analyzed using the International Physical Activity Questionnaire (IPAQ) – short form, and the patients were classified as being inactive, being minimally active, or participating in health enhancing physical activity (HEPA; i.e., highly active) [30, 31].

In the morning, after the participant had fasted for 12 h, a 10 mL blood sample was collected by a trained nurse using disposable material. All the samples were then centrifuged at 2000 rpm for 10 min at room temperature to test high-sensitivity C-reactive protein (hs-CPR), erythrocyte sedimentation rate (ESR), hemoglobin A1c, fasting insulin and glucose levels, total cholesterol and its fractions and triglycerides. HOMA-IR it was also used to calculate insulin resistance. All data were analyzed using SPSS software, version 19.0. The Kolmogorov-Smirnov test was used to evaluate the normality of distributions.

Descriptive analysis was expressed as mean, standard deviation, and frequency (%). The sample was calculated by using chi-square test. A power of 80% power and a significance of 5% were used, and the required sample size was determined to be 97. Inferential statistics included Student’s t-test to compare means of numeric variables that were normally distributed. The Pearson correlation coefficient was used to test associations among continuous variables, including PASI, DAS-28, BASDAI, HAQ, number of swollen joints (NSJs) and number of tender joints (NTJs), age, weight, height, BMI, and waist circumference. To perform the multiple regression analyses, a model was created for each outcome: PASI for skin disease activity and MDA for joint disease activity. Both PASI and MDA were considered as dependent variables, while biochemical (fasting glucose, HOMA-IR, cholesterol, triglycerides), body composition measurements (waist circumference, lean mass, fat mass, BMI), dietary and activity habits, pharmacological treatments, and clinical conditions were considered independent variables. The final regression model was adjusted for sex, weight and age. The level of significance was set as *p* < 0.05.

The study was approved by the Ethics Committee of Research from Federal University of Sao Paulo (CAAE: 00591412.5.0000.5505).

## Results

Patients with PsA were equally distributed according to sex (54.6% females), and there were more adults (68%) than elderly with long-term disease. Most PsA patients were inactive (35.1%) or minimally physically active (39.2%), according to the IPAQ. Approximately 60% of women were postmenopausal (60.4%), and almost 20% were taking hormone replacement therapy (data not shown).

More than 60% had skin involvement primarily, and only 14% had arthritis as the initial manifestation. Almost 25% of the sample had both manifestations concomitantly. Methotrexate (MTX) was used by the majority of patients as monotherapy or in combination with TNF-blockers. Approximately 20% of patients were taking TNF-blockers, and less than 10% of the sample was using nonsteroidal anti-inflammatory drugs (NSAIDs) or glucocorticosteroids. There was a high prevalence of obesity, according to BMI, and abdominal fat tissue excess (Table 1).

**Table 1:** Characteristics of patients with psoriatic arthritis

	*N* (%)	Mean (SD)
Age (years)		53.12 (13.10)
Sex (Female)	53 (54.6)	
Weight (kg)	–	77.93 (15.98)
Height (cm)	–	159.1 (20.2)
Body Mass Index (kg/m^2^)	–	30.50 (5.73)
Waist circumference (cm)	–	103.13 (13.26)
Female	–	104.44 (13.40)
Male	–	101.43 (13.49)
Time of Disease (years)		12.29 (14.42)
Skin	–	19.27 (15.26)
Joint	–	12.98 (14.43)
Skin activity		
PASI	–	3.61 (6.84)
BSA	–	4.22 (9.31)
Joint activity		
Arthritis	72 (74.2)	
Enthesitis	30 (30,9)	
Dactylitis	8 (8.72)	
Axial	34 (35,1)	
DAS28-ESR	–	3.54 (1.38)
Remission	21 (22.1)	–
Low	17 (17.9)	–
Moderate/Severe	57 (60.0)	–
DAS28-CRP		3.05 (1.31)
Remission	34 (36.2)	–
Low	21 (22.3)	–
Moderate/Severe	39 (41.5)	–
BASDAI	–	3.15 (2.06)
< 4	47 (52.8)	–
≥ 4	42 (47.2)	–
HAQ	–	0.92 (0.69)
MDA	22 (23.4)	
Psysical Activity (IPAQ)		
Inactive	60	
Minimally active	27	
Sufficiently active	1	
Active	0	
Very active	0	
Drug therapy		
GCs	11 (11.5)	
NSAIDs	4 (4.1)	
Synthetic DMARDs monotherapy	31 (31.9)	
Synthetic DMARDs combination therapy	10 (10.3)	
TNF-blockers	17 (17.5)	
Infliximab	4 (4.1)	
Etanercept	5 (5.2)	
Adalimumab	8 (8.2)	
Synthetic and biological DMARDs	14 (14.4)	
Comorbidities		
Diabetes, *n* (%)	20 (20.6)	
Hypertension, *n* (%)	45 (46.4)	
Dyslipidemia, *n* (%)	43 (44.3)	
Concomitant Medications		
Insulin, *n* (%)	7 (7.2)	
Statins, *n* (%)	33 (34.0)	
Antidiabetic, *n* (%)	20 (20.6)	
Antihypertensive, *n* (%)	46 (47.4)	

Results shown as absolute values and respective percentage or mean and standard deviation (SD). *GCs* glucocorticoids, *NSAIDs* non-steroidal anti-inflammatory drugs, *MTX* methotrexate, *LEF* leflunomide, *CsA* cyclosporine A, *TJN* tender joint number, *SJN* swollen joint number, *DMARDS* Disease-modifying antirheumatic drugs, *TNF* Tumoral Necrosis Factor

After sex and age-adjustments, there was a high prevalence of abdominal fatness (android pattern) in both male and female PsA patients, but no significant lean or bone mass impairment was observed (Table 2). More than 90% of PsA patients, regardless of sex, had excess adipose tissue (FMI), 5.2% had sarcopenia and 3.1% had sarcopenic obesity, according to DXA measurements and NHANES III cutoffs. Comparing FMI and BMI, there was a divergence between these two measurements in almost 20% of patients in the classification of fat excess. PsA patients also had a high rate of MetS (54.6%), hypertension (46.9%), and dyslipidemia (44.3%).

**Table 2:** Body composition measurements in patients with PsA, according to sex

	Male (*N* = 43**)	Female (*N* = 53)
Waist Circumference 15		
Adequate, *N* (%)	7 (7.2)	3 (3.09)
High, *N* (%)	37 (38.1)	50 (51.55)
Lean mass		
Total (kg)	51.8 ± 11.1	39.19 ± 7.14
Appendicular (kg)	24.49 ± 4.34	17.05 ± 2.94
ASMI (kg/m^2^)	8.54 ± 1.29	6.94 ± 0.87
Fat mass		
Total (kg)	24.8 ± 9.51	33.75 ± 11.13
Total (%)	30.85 ± 8.40	45.17 ± 8.64
FMI (total fat mass/height^2^)	8.98 ± 3.87	13.93 ± 4.68
Android (%)	41.78 ± 10.05	50.74 ± 10.01
Gynoid (%)	32.95 ± 7.10	48.84 ± 8.75
A/G Ratio	1.21 ± 0.23	1.04 ± 0.16
A/G Ratio > 1*, *N* (%)	37 (84.1)	34 (64.2)
Bone Mass		
BMC (g)	2.82 ± 0.45	2.22 ± 0.38
BMD (g/cm^2^)	1.20 ± 0.10	1.12 ± 0.21
Fat Mass Index(1), *N* (%)		
Severe fat deficit	1 (2.3)	0 (0)
Moderate fat deficit	1 (2.3)	0 (0)
Mild fat deficit	0 (0)	0 (0)
Normal	3 (7.0)	1 (1.9)
Excess fat	8 (18.6)	5 (9.4)
Obese class I	20 (46.5)	14 (26.4)
Obese class II	6 (14)	21 (39.6)
Obese class III	4 (9.3)	12 (22.6)

*ASMI* Appendicular skeletal muscle mass index, *FMI* Fat Mass Index, *A/G* Android/Gynoid, *BMC* Bone Mineral Content, *BMD* Bone Mineral Density. *A/G Ratio > 1 = Android pattern. **Missing anthropometric data and total body DXA measurements: 1 patient

Regarding dietary intake, there was a high average energy consumption, especially in men, but there was no significant difference in macro or micronutrient intake, according to sex. Furthermore, low consumption of fiber was found, as well as consumption of sodium above the international recommendations (Table 3). The ω-6/ ω-3 ratio was 5.8/1.

**Table 3:** Daily food intake of patients with psoriatic arthritis, according food intake records

Nutrient	Mean (SD)			Recommendation (Male/Female)
	Total	Male	Female	
Energy (Kcal)	1955.9 (730.0)	2291.7 (649.7)	1683.5 (681.0)	–
Carbohydrate (g)	257.9 (48.2)	259.7 (55.3)	256.3 (42.1)	130/130
Protein (g)	85.9 (17.4)	88.9 (17.0)	83.5 (17.5)	56/46
Lipid (g)	63.0 (17.1)	59.8 (14.9)	65.5 (18.3)	–
SFA (g)	21.6 (9.3)	19.7 (6.3)	23.2 (11.0)	–
MUFA (g)	12.0 (5.4)	11.9 (5.9)	12.1 (5.0)	–
PUFA (g)	4.19 (2.72)	4.14 (2.23)	4.22 (3.06)	–
Cholesterol (mg)	222.5 (93.6)	217.4 (87.6)	226.5 (98.8)	**
Trans fat (g)	0.99 (0.62)	0.94 (0.24)	1.03 (0.08)	**
Sugar (g)	88.2 (39.5)	88.7 (51.4)	87.8 (27.1)	143/105
Fibers (g)	2.38 (0.38)	2.40 (0.36)	2.36 (0.39)	30/ 21
Omega 3 (g)	0.37 (0.12)	0.37 (0.11)	0.36 (0.13)	1.5–3.0/ 1.1–2.2
Omega 6 (g)	2.14 (1.49)	2.13 (1.55)	2.15 (1.45)	12.7–25.4/ 9.3–18.7
Sodium (mg)	2578.6 (784.4)	2589.5 (925.7)	2569.6 (657.0)	1300/1300

Results shown as mean and standard deviation (SD). *SFA* Saturated fatty acid, *MUFA* monounsaturated fatty acid, *PUFA* Polyunsaturated fatty acid, *REq* Retinol equivalent, Energy adjusted (2). Recommendation according Estimated Average Requirements (3)

**As low as possible while consuming a nutritionally adequate diet

Biochemical analysis showed that insulin resistance (HOMA-IR), fasting blood glucose levels, and hemoglobin A1c were above the reference values (Table 4). Aproximately 30% of the sample had values above the recommended values and were being treated for glucose intolerance (20.6% metformin and 7.2% insulin therapy). However, almost 70% of subjects with abnormal HOMA-IR had not yet been diagnosed with diabetes mellitus. In contrast, serum cholesterol levels were adequate in most patients. Statins or fibrates were being used by 34% of the patients.

**Table 4:** Biochemical profile of patients with PsA

	Mean ± SD
Fasting glucose levels (mg/dL)^a^	102.90 ± 34.75
Hemoglobin A1c (%)^b^	6.14 ± 1.27
Insulin (U)^c^	16.88 ± 14.08
HOMA-IR^d^	4.59 ± 5.19
Total Cholesterol (mg/dL)^e^	196 ± 42.4
LDL-Cholesterol (mg/dL)^f^	121.1 ± 37.6
HDL-Cholesterol (mg/dL)^g^	47.7 ± 11.36
Triglycerides (mg/dL)^h^	138.87 ± 86.39

Cutoff points: a) 99 mg/dL. b) > 6%. c) > 30 U. d) > 2.7(4). e) > 200 mg/dL. f) > 130 mg/dL. g) < 40 mg/dL ♂ and < 50 mg/dL ♀. h) > 150 mg/dL

There was a slightly moderate correlation between the values of joint disease activity and body composition measurements, including DAS28-ESR and FMI (r = 0.33, *p* = 0.001), bodily fat (r = 0.40, *p* < 0.001) and BMI (r = 0.20, *p* = 0.049). On the other hand, there was a negative correlation between DAS28-ESR and appendicular skeletal lean mass index (r = − 0.38, *p* < 0.001). Similarly, a positive correlation was found between DAS28-CRP and fat, as well as FMI (r = 0.27, *p* = 0.008), body fatness (r = 0.27, p = 0.008), BMI (r = 0.26, *p* = 0.01) and waist circumference (r = 0.27, *p* = 0.009) (data not shown).

No significant correlation was found between skin activity and body composition measurements (data not shown), although there was a significant correlation between the PASI and the serum cholesterol levels (r = 0.30; *p* = 0.003 and r = 0.28; *p* = 0.006, for total and LDL cholesterol, respectively). However, it is important to mention that the mean PASI was low in this population. Thus, the correlations related to skin activity might be more relevant in patients with more severe psoriasis.

Patients in remission had significantly higher lean mass than those with active arthritis. Nonetheless, those with more severe joint activity had higher FMI and fat intake (Table 5). Unexpectedly, no correlation was found between MDA and body composition measurements, food intake, or biochemical indexes. On the other hand, skin disease activity was more severe in patients with increased consumption of trans fat and sodium and lower ω-6 intake than in patients in remission (Table 6). After multiple statistical adjustments, including adjustments for sex, BMI and age, the final model of multivariate regression model showed that total body fat (R^2^ = 0.065, *p* = 0.02) and insulin resistance (R^2^ = 0.069, *p* = 0.016) were significantly associated with joint activity. However, no variable could explain the outcome related to skin activity.

**Table 5:** Joint activity and body composition measurements in PsA patients

	DAS28-ESR			
Body Composition	Severe (> 5.1)	Moderate (3.2–5.1)	Low (2.6–3.2)	Remission (< 2.6)
ASMI (kg/m^2^)	17.74^a^	19.54 ^a^	19.57 ^a^	23.91 ^b^
Total body fat mass (kg)	45.53 ^a^	40.46 ^ab^	37.05 ^ab^	31.78 ^b^
FMI (kg/m^2^)	14.45 ^a^	12.53 ^ab^	10.22^b^	9.69^b^

*ASMI* Appendicular skeletal muscle mass index, *FMI* Fat mass index, *ANOVA* Tukey Test, Same letters in the same line *p* > 0.05; different letters in the same line *p* < 0.05

**Table 6:** Associations between skin activity and food intake in patients with PsA

Food Intake	PASI		
	Severe (> 5.0)	Moderate (0.1–4.9)	Remission (0)
Trans fat (g)	**1.59**^**a**^	1.01^b^	0.91^b^
Sodium (mg)	**2921.82**^a^	2053.96^b^	2588.02^b,a^
Omega 6 (g)	**1.65**^a^	3.12^b^	2.11^a,b^

ANOVA and Tukey test; same letters in the same line *p* > 0.05; different letters in the same line *p* < 0.05

The boldface indicates significative association (*p* > 0.05) between trans fat intake and severe and moderate PASI and between sodium and moderate and severe PASI.

## Discussion

Our results demonstrated that patients with active PsA had high rate of obesity, metabolic syndrome and adiposity (FMI), as well as high fat intake and insulin resistance, suggesting that these aspects share a possible harmful link with the association between disturbed lipid and glucose metabolism and skin and joint disease. It is worth emphasizing that the fat consumption and cholesterol levels might be more associated with skin activity, whereas the excess of total and abdominal fat mass and lower lean mass were more related to joint activity.

This close relationship between fat and skin and joint disease may be explained by inflammation itself. The increase in macrophages and other immune cells in psoriatic lesions and the synovio-entheseal complex would promote complex metabolic changes in the liver and adipose tissue, especially insulin resistance, as well as increased release of TNF-α and lower production of adiponectin [8]. Furthermore, patients with PsA and psoriasis share some other pathophysiological characteristics (neoangiogenesis, insulin uptake, adipogenesis, lipid metabolism, and immune and epidermal proliferation) [6–8] and genetic aspects, such as peroxisome proliferator activated receptor (PPAR) polymorphism. Altogether, they have been considered triggers of inflammatory disorders and immune cell activation [32, 33].

PPARs are ligand-dependent transcription factors, that are activated by fatty acids and their derivates and control the inflammatory response. PPARs is are an important link between the alterations of lipid and glucose metabolism and innate immunity, and are divided into tree subtypes: PPAR-α, PPAR-Β, PPAR-γ [33].

PPAR-α modulates inflammation in macrophages preventing atherogenesis and modulating cholesterol transport. The activation promotes glucose accumulation, the synthesis of ketone bodies and fatty acid oxidation. PPAR-Β decreases the production and activation of proinflammatory cytokines related to insulin resistance in adipocytes and improves steatosis in the liver. PPAR-γ is mediated by adiponectin and protects against vascular injury. Therefor, there is an alteration in PPAR expression in psoriasis and PsA, contributing to the systemic inflammation in both diseases and the alteration of fat and glucose metabolism [33].

It seems that the oral treatment of PPAR syntetic ligand decreases inflammatory cytokines and suppresses angiogenesis, and PPAR syntetic ligand has been used in the treatment of metabolic syndrome, diabetes and dyslipidemia. Pilot studies have also shown that oral administration of the PPAR agonist improved cutaneous symptoms of PsA and psoriasis [33].

Higher cardiovascular risk in patients with PsA can also be attributed to the combination of skin and joint inflammation, releasing higher amounts of pro-inflammatory cytokines, such as tumor necrosis factor (TNF)-α, interleukin (IL)-6, IL-17 and IL-23 [34].

Recent studies have shown the importance of the IL-23/IL-17 axis pathway for the pathogenesis of chronic and autoimmune diseasse, including PsA, indicating an interaction between components of the innate and adaptive immune system. While IL-23 is critical in the pathogenesis of autoimmunity and producing myeloid cells, granulocytes, macrophages, and mast cells, IL-17 contributes to Th17- and IL17-producing cytotoxic (CD8+) T cells [35, 36].

Adiposity and insulin resistance (IR) also contributes to immunity and inflammation. The adipose tissue produces adipokines, especially leptin, adiponectin, resistin, and visfatin [37].

Leptin is up-regulated by inflammatory mediators and promotes the increase of TNF, IL-12, IL-6 and others inflammatory cytokines. The hyperleptinemia and leptin resistance, a common scenario on obesity, leads to a reduction in adipose tissue-infiltrating regulatory T cells (T_reg_), amplifying local inflammation. T_reg_ cells are a subcategory of CD4+ CD25+ T cells, critical mediators of immune tolerance [37].

Resistin and visfatin are up-regulated with the increase of pro-inflammatory cytokines. While resistin is associated with glucose metabolism, visfatin has an important role in the development of B and T lymphocytes and it operates as a chemotactic factor for lymphocytes and monocytes. In contrast, adiponectin acts decreasing TNF and IL-6, and increasing interleukin-1 receptor associated kinase 1 (IRAK-1) in macrophages, monocytes, and dendritic cells, implying negative feedback between adiponectin and proinflammatory cytokines [37].

Along these lines, it seems that there is a correlation between levels of IL-17, IL-23 and increased weight adipose tissue. Adiposity is an important source of proinflammatory mediators and infiltrating immune cells, representing a possible cellular source of IL-17 and IL-23 in obese patients [37].

Another aspect that is also related to the higher incidence of atherosclerosis in PsA patients than in he general population is related to the increased prevalence of MetS and each of its components, such as obesity, hypertension, diabetes mellitus and dyslipidemia [34].

Regarding food intake, our data showed that patients with PsA had a high consumption of energy, saturated fat and sodium and low intake of ω-6, ω-3 and fiber. It has been well established that inadequate consumption of these nutrients and a hypercaloric diet are associated with hypertension, diabetes, dyslipidemia and metabolic syndrome [38], conditions which are conditions with high prevalence in PsA patients.

The energy consumption and low fiber intake were quite similar to the findings in the Brazilian population [39]. However, the ω-6/ω-3 ratio was lower than recommended by the World Health Organization [40]. This finding is important to mention because higher ω-6/ω-3 ratio is associated with inflammatory disorders and cardiovascular diseases [41]. Although we did not find any correlation with carbohydrate intake, it was observed that patients with severe skin activity had higher intakes of sodium and trans fat and reduced consumption of ω-6 than patients with moderate activity or without skin lesions. These results highlight that these nutrients may be associated with disease activity, as reported by Medeiros and Sittart [16].

Despite this data seems controversial whereas ω-6 fatty acid is commonly associated with increased inflammation, it is relevant to consider that ω-6 does not produce only pro-inflammatory eicosanoids, but also lipid mediators that play an important role in inflammation resolution. Evidently, in healthy human adults, the increased intake of arachidonic acid (ARA) or linolenic acid (LA) does not increase the many of the levels of the inflammatory markers. Thus, the ratio of omega-3 and omega-6 polyunsaturated fatty acids (PUFAs) seems to be the best option to evaluate in the context of inflammation [42].

Furthermore, the body and abdominal (android pattern) adiposity excess in PsA patients was significantly higher than the normative data from the female Brazilian population [20]. Our data demonstrated that BMI was not fully able to identifying excess fat, unlike DXA measurements. Using different methodologies, such as bioimpedance and plethysmography, previous studies also found similar results [4, 43]. Unexpectedly, we found sarcopenia in only 5% of our sample and binomial obesity-sarcopenia was also only evidenced in 3% of PsA patients, in contrast to the findings of another authors [4, 44].

Beyond inadequate food intake and body composition changes, there was a higher frequency of peripheral insulin resistance, as well as a significant relationship with joint activity, highlighting a high prevalence of undiagnosed diabetes/insulin resistance in patients with PsA. First, this finding was attributed to obesity itself. However, merging our data, we suggest that these conditions might share pathophysiological phenomena and are not derived from each other. The PPAR-γ polymorphisms may be a canonical pathway to both disorders [32, 33].

Supporting this hypothesis, we were able to show a significant correlation between psoriatic disease activity and body composition measurements, including the negative association with lean mass and the positive association with fat mass. In addition, we demonstrated a positive association between skin activity and total and LDL-cholesterol serum levels as well as higher trans fat intake and lower ω-6 consumption. Some studies have demonstrated that weight loss is associated with lower disease activity and improved drug response [45]. Altogether, adipose tissue is a relevant player for modulating skin and joint activity in PsA patients.

Excess adipose tissue is closely associated with a higher risk for MetS and is likely related to PsA, according to our data. Some authors have demonstrated that, in early adulthood, each 1-unit increase in BMI the risk of PsA by 5.3% and that obesity increases this risk three times, suggesting that excess body weight can be a predisposing factor to PsA. Obesity in PsA patients seems to be associated with reduced probability of achieving minimal disease activity and increases the cardiovascular risk. In addition, patients with PsA have some limitation in performing exercises, especially patients with moderate or severe disease activity, increasing sedentarism, similar to what was shown by our data [3]. Altogether, these aspects are associated with systemic inflammation, liver steatosis, insulin resistance, lipid oxidation, atherosclerosis and cardiovascular risk.

Hence, the implementation of nonpharmacological strategies such as weight loss (10% of total body weight), regular physical activity, and counseling about protein, carbohydrate, and fat intake could contribute to drug intervention outcomes and the clinical prognosis of these patients. However, prospective observational studies and randomized trials are needed to demonstrate the true impact of body composition changes and dietary style on disease activity in patients with PsA.

Despite the sample size seems a limitation, the number was calculated by using statistic analyses. All patients of the four biggest centers of rheumatology in Sao Paulo state was recruited to participate of this study. Initially, 140 patients were recruited, but thirty-three did not fill the inclusion criteria. We had a total of 97 patients included in this study, same number calculated by the Chi-square test.

This study had some positive points, such as a comprehensive and integrated view regarding the relationships among food intake, inflammation, body composition measurements, and metabolic changes in PsA patients. However, it also has some limitations, including the lack of a control group, the daily variation in food intake and the exclusion of very obese patients (more than 135 kg) due to the DXA weight limit.

We are aware of the limitations of DAS28 in evaluating PsA joint activity. Although there are many tools to measure disease activity in these patients, including PASDAS, DAPSA and CPDAI, none of them have been used in a majority of clinical trials or in epidemiological studies [46]. In addition, the MDA has been recommended by the EULAR (European League Against Rheumatism) and GRAPPA (Group for Research and Assessment of Psoriasis and Psoriatic Arthritis) to better evaluate the skin and joint outcomes in these patients, and because of that, this instrument it was chosen in our study [29, 47]. More recently, Coates et al. demonstrated a close relationship between MDA and other composite indexes (above 0.9), including PASDAS and CPDAI [48].

## Conclusion

Thus, patients with active PsA have higher prevalence of obesity and adiposity and excess total and abdominal fat, as well as MetS, insulin resistance and inadequate fat food intake and higher total and LDL-cholesterol serum levels, suggesting a harmful link among lipid and glucose metabolism and joint and skin disease.

## Data Availability

Data supporting our findings can be found at Rheumatology Division in Federal University of São Paulo (715 Napoleao de Barros St., Vila Clementino, São Paulo/ SP, Brazil. ZIP code: 04024–002).

## Abbreviations

ALM: Appendicular Lean Mass
ARA: Arachidonic Acid
BASDAI: Bath Ankylosing Spondylitis Disease Activity Index
BASI: Body Surface Area
BC: Body Composition
BMI: Body Mass Index
CASPAR: Classification Criteria of Psoriatic Arthritis
CD8: Cytotoxic
CPDAI: Composite Psoriatic Disease Activity Index
CPR: C-reactive Protein
CVD: Cardiovascular disease
DAPSA: Disease Activity Index for Psoriatic Arthritis
DAS28: Disease Activity Score
DRI: Dietary Reference Intake
DXA: Dual X-ray-absorptiometry
ESR: Erythrocyte Sedimentation Rate
EULAR: European League Against Rheumatism
FMI: Fat Mass Index
FR: Food-record
GRAPPA: Group for Research and Assessment of Psoriasis and Psoriatic Arthritis
HAQ: Health Assessment Questionnaire
HDL-C: high-density lipoprotein
HEPA: Health Enhancing Physical Activity
HOMA-IR: Insulin Resistance
Hs-CRP: High-Sensitive C-Reactive Protein
IDF: International Diabetes Federation
IL: Interleukin
IPAQ: International Physical Activity Questionnaire
IRAK-1: Increasing Interleukin-1 receptor associated kinase 1
ISCD: International Society Clinical Densitometry
LA: Linolenic Acid
LDL: Low-density lipoprotein
MDA: Minimal Disease Activity
MetS: Metabolic Syndrome
NSAIDs: Non-steroidal Anti-inflammatory drugs
NSJ: Number of Swollen Joints
NTJ: Number of Tender Joints
PASDAS: Psoriatic Arthritis Disease Activity Score
PASI: Psoriasis Area Severity Index
PPAR: Peroxisome Proliferator-Activated Receptor
Ps: Psoriasis
PsA: Psoriatic Arthritis
PUFAs: Polyunsaturated Fatty Acids
TNF-α: Tumor Necrosis Factor
T_reg_: Regulatory T cells
WHO: World Health Organization
ω-3: Omega 3
ω-6: Omega 6
